# Acute toxicity profile in prostate cancer with conventional and hypofractionated treatment

**DOI:** 10.1186/1748-717X-8-94

**Published:** 2013-04-21

**Authors:** Gustavo Arruda Viani, Lucas Bernardes Godoy da Silva, Bruna Bueno da Silva, Yuri Bonicelli Crempe, Vinicius Spazzapan Martins, Ricardo Jose Rambaiolo Ferrari, Mariana Colbachini Pólo, Bruno Thiago Rossi, Elton Suguikawa, Giseli Correa Zulliani, Eduardo Jose Stefano

**Affiliations:** 1 Department of Radiation Oncology, Marilia Medical School, Marília, São Paulo, Brazil; 2 Marilia Medical School, Marília (FAMEMA), São Paulo, Brazil; 3 Department of Radiation Oncology, Marilia Medical School, Maria Cecilia Alves, 192, Marilia, São Paulo, Brazil

**Keywords:** Acute toxicity, Prostate cancer, Hypofractionation, Conformal radiotherapy

## Abstract

**Purpose:**

To compare the acute toxicities in radical treatment of prostate cancer between conventional schedule (C-ARM) with 78 Gy/39 fractions and hypofractionation conformal treatment (H-ARM) with 69 Gy/23 fractions.

**Methods and material:**

This prospective double arm study consisted of 217 patients with prostate cancer, 112 in H-ARM and 105 in C-ARM arm. C-ARM received conventional six- field conformal radiotherapy with 78 Gy in 39 fractions while H-ARM received hypofractionation with 69 Gy in 23 fractions. Weekly assessment of acute reactions was done during treatment and with one, and 3 months using RTOG scale. Univariated analysis was performed to evaluate differences between the incidences of acute reaction in the treatment arms. Variables with p value less than 0.1 were included in the multivariated logistic regression.

**Results:**

There was no difference between H-ARM versus C-ARM for severity and incidence in genitourinary (GU) and gastrointestinal (GI) acute toxicity. During the treatment comparing H-ARM with C-ARM no differences was observed for GI toxicity (grade 0–3; H-ARM = 45.5%, 34%, 18.7% and 1.8% versus C-ARM = 47.6%, 35.2%, 17.2% and 0). For acute GU toxicity no difference was detected between H-ARM (grade 0–3; 22.3%, 54.5%, 18.7% and 4.5%) and C-ARM (grade 0–3; 25.8%, 53.3%, 17.1% and 3.8%).

At the 3- months follow-up, persistent Grade > =2 acute GU and GI toxicity were 2.5% and 1.8% in H-ARM versus 5.7% and 3% in C-ARM (p > 0.05). In univariated and multivariated analyses, there was not any dosimetric predictor for GI and GU toxicity.

**Conclusions:**

Our data demonstrate that hypofractionated radiotherapy achieving high biological effective dose using conformal radiotherapy is feasible for prostate cancer, being well tolerated with minimal severe acute toxicity.

## Introduction

In the last decades, it has been shown that exists a dose–response relation between the prostate cancer biochemical control and the total dose of radiotherapy delivered [1]. Evidences have also been growing from experimental and clinical studies that the α/β ratio of the linear-quadratic formulation for prostate cancer might be between 1.5 and 1.85 Gy [2-4]. This low α/β ratio suggests that prostate cancer has high sensitivity to dose per fraction, which suggests that a hypofractionation, with a large radiation dose delivered in a smaller number of fractions, might be more advantageous when compared to other type of cancer cells. On the other hand, the α/β ratio of the rectum is as important as that of prostate cancer for exploring which hypofractionation regimens will be most beneficial. Although α/β ratio for the rectal wall is not known precisely, animal studies suggest α/β ratio for the rectum of 4–6 G [5] . If the α/β ratio for rectum is higher than that for prostate, theoretically, larger hypofractionated doses could be given with larger clinical gains within the same or lower complication rates [6].

Although the hypofractionation schedule for prostate cancer appears more attractive than conventional fractionation, the experience of using hypofractionation with total equivalent doses of 78–80 Gy has been limited.

These limited data are from a randomized Phase III trial comparing a conventional fractionation regimen of 80 Gy given in 2-Gy fractions with a hypofractionation regimen of 62 Gy given in 20 fractions of 3.1 Gy/d [7], a nonrandomized study of hypofractionation vs. conventional fractionation delivered using 3D conformal radiotherapy technique [8], and a few other Phase I-II reports using image guide radiotherapy (IGRT) or intensity-modulated radiotherapy (IMRT) [9-11].

In order to compare two-fractionation regimens of radiotherapy in prostate cancer patients, we compared a high dose hypofractionation schedule (69 Gy/ 23 fractions) with conventional fractionation (78 Gy/39 fractions). This report summarizes the acute genitourinary and gastrointestinal side effects for all patients included in our prospective nonrandomized study, comparing conventional or hypofractionated RT.

## Material and methods

It is a prospective study conducted after the approval of the institutional review board. The study population consisted of 217 patients with localized prostate cancer, who were treated between November 2009 and January 2011, with patients selected into two arms. Patients in C-Arm received conventional radiotherapy and those in H-Arm received hypofractionated radiotherapy, both treatments with conformal technique.

### Evaluation

The pretreatment evaluation consisted of a full history, with special emphasis on pretreatment urinary and rectal symptoms, and a physical examination. The prognostic groups were defined as follows: low risk, Stage T1-T2a, Gleason score <7, and initial prostate-specific antigen (iPSA) level <10 ng/mL; intermediate risk, Stage T1-T2b, Gleason score <7, and iPSA level of 10–19.9 ng/mL or Stage T1-T2b, Gleason score 7, and iPSA <20 ng/mL; and high risk, Stage T3, Gleason score 8–10, or iPSA >20 ng/mL. Patients with metastases were excluded this trial.

### Selection for treatment arms

Patients were selected for treatment arms according to their convenience. This bias was permitted, because the most patients came from long distances to treat. The groups were balance to achieve similar distribution between the treatment arms. If a patient was chosen to be treated in the H- Arm, the next patient automatically was allocated in C-arm.

### Treatment

For each patient, a conformal radiotherapy plan consisting of six 6-MV photon beams was used to deliver 69 Gy in 23 daily fractions of 3 Gy or 78 Gy in 39 fractions of 2 Gy, prescribed at the isocenter. No patient was treated with Intesity Modulated Radiotherapy (IMRT) and without any fiducial. This dose was used because, according to the linear quadratic formula, it is biologically equivalent to 87.8 Gy in 2 Gy fractions or 90 Gy in 50 fractions assuming an α/β ratio of 1.8 and 1.5 Gy, respectively. All patients underwent a treatment planning CT (5-mm slice thickness) in the supine position with a triangle sponge placed under their knees. Patients were advised to have a comfortably full bladder and an empty rectum at time of computed tomography (CT) simulation. The prostate, seminal vesicles base, whole rectum, bladder, femoral heads, and penile bulb were contoured in all patients. The clinical target volume was the prostate plus seminal vesicles base; no patient had the pelvis included in the clinical volume. The planning target volume consisted of the clinical target volume plus a uniform 10-mm margin in all directions, excluding the rectal margin (7 mm). All fields were treated daily. Single exposure portal films were obtained previous to the first fraction and weekly thereafter. Dose–volume histograms (DVHs) were calculated for a rectal volume encompassing the organ from anal merge to the retosigmoid transition. Rectal DVH constraints were V60 Gy < = 50%, V65 Gy < = 35%, V 70Gy < = 25% and V 75Gy < = 15% for C-Arm. In the H-Arm, DVHs were calculated from the standard 2-Gy fractionation equivalent to the doses of 3 Gy by fraction (assuming α/β = 3 Gy). Table 1 describes the rectal and Bladder DVH constrains used in H-Arm and C-Arm. The software used was Eclipse version 8.6, Varian Medical System, Inc, Palo Alto, USA. Patients considered as intermediate or high risk were treated with neoadjuvant, comcomitant and adjuvant androgen blockage. The intermediate risk received 6 months and high risk 24 months of androgen blockage. The androgen blockage consisted of a mensal intramuscular injection of acetate of goserelin of 3.6 mg.

**Table 1:** Dose constrains

**Variables**	**C-Arm**	**H-Arm**
**PTV**	95% of PTV78 to receive 78 Gy	95% of PTV69 to receive 69Gy
	(by definition)	(by definition)
**Rectum**	V60 Gy < = 50%	V50Gy < = 50%
	V65 Gy < = 35%	V54Gy < = 35%
	V70Gy < = 25%	V58Gy < =25%
	V75Gy < = 15%	V62Gy < = 15%
**Bladder**	V65Gy < = 50%	V54Gy < = 50%
	V70Gy < = 35%	V58Gy < = 35%
	V75Gy < = 25%	V62Gy < = 25%
	V80Gy < = 15%	V67Gy < = 15%
**Femurs**	Maximal dose < = 55 Gy	Maximal dose < = 46 Gy

### End points

The primary study outcome was acute treatment reactions from the beginning of treatment to 3 months after the end of treatment. Patients were seen weekly, or as required, during treatment by a radiation oncologist. Pre-existent urinary or rectal disorders, such as dysuria, pollakiuria, stress incontinence, hemorrhoids, and so forth, were assigned a grade complication if RT had exacerbated the baseline dysfunction. Acute gastrointestinal (GI) (retite, diarrhea, tenesmus and fecal incontinence) and genitourinary (GU) toxicity (dysuria, urinary frequency, retention, hematuria and urinary incontinence) were prospectively assessed and graded according to the Radiation Therapy Oncology Group scoring system for the rectum and bladder.

### Statistical analysis

The association between the two groups was determined through bivariate analysis using Pearson’s chi square test or Fisher test, when necessary. To compare continuous variable the student T test was used. Two sided p value was calculated and any difference with a p value < 0.05 was considered significant. The dosimetric parameters such as; rectal, PTV and bladder volume were extracted from the treatment plan as well as age and ADT. These variables were tested whether they were related to the probability of having > = Grade 2 RTOG toxicity. Univariated analysis and multivariated logistic regression were performed to evaluate differences between the incidences of acute reaction in the treatment arms. All statistical analysis was performed using SPSS (Statistical Analysis Systems software), version 19.

## Results

A total of 217 patients (112 H-Arm and 105 C-Arm) treated on protocol from December 2009 to January 2011 were prospectively analyzed in this study. Baseline characteristics for all patients are shown in Table 2. The median age at last follow-up was 72 and 71 years for C-Arm and H-Arm, respectively. The follow-up for all patients was 3 months. Acute toxicity was weakly assessed during treatment and as needed by the patient before the regular follow-up. The majority of the patients tolerated the treatment well without major acute GI or GU side effects during the treatment (47.6% in C-Arm and 45.5% in H-arm for GI toxity and 25.8% in C-Arm versus 22.3% H-Arm for GU toxicity), as described in Table 3. The maximum acute GI and GU toxicities are shown for the whole group and for each fractionation schedule in Table 3. There was no apparent difference in any acute toxicity when compared the H-Arm to the C-Arm (p >0.05). During the treatment there were 23% of those in H-Arm and 19% in C - Arm taking medication to improve urinary function (e.g., alpha blocker, antispasmodic, analgesic such as Pyridium). At 3 months of follow up, only 2 patients in H-Arm (1.8%) experiencing an acute GI toxicity score of 2 or more. Otherwise, in C – Arm 4 patients (3.8%) experienced an acute GI toxicity score of 2, and 2 patients (1.9%) experienced an acute GU toxicity score of 3. Three patients in each arm developed acute GU toxicity Grade 3 (2.5% in H-arm and 3% in C-arm), as seen in Table 4. With 3-month follow up there were 15% and 13% in H-Arms and C-Arms taking medication to improve urinary function; the difference was not significant.

**Table 2:** Patients characteristics

**Characteristic**	**C-Arm**	**Hypo-Arm**	**P value**
**Patients (n)**	105	112	
**Age (median)**	72	71	0.913
**Baseline Gleason score**			
>7	40	42	0.384
<=7	65	70	0.765
**Initial PSA level (ng/mL)**			
Mean	8.6	9.2	0.682
Range	0.72- 58.4	0.59 – 62.8	
**Risk stratification**			
Low risk	38	35	0.424
Intermediate Risk	44	40	0.485
High Risk	40	30	0.827
**Androgen treatment**	84	70	0.938
**Follow-up (mo)**	3 months	3 months	
**Mean rectal volume (cm3)**	60.8 + −2.9	59.7 + −2.7	0.512
**Mean bladder volume (cm3)**	262.0 + −16.5	258.9 + − 17.7	0.239
**PTV Mean total volume (cm3)**	167.5 + − 30.5	162.8 + − 31.2	0.114

**Table 3:** Incidence of maximum acute RTOG toxicity during the treatment

**Characteristic**	**C-Arm**	**Hypo-Arm**	**P value**
**GI toxicity**			
Grade 0	50 (47.6)	51 (45.5)	0.643
Grade 1	37 (35.2)	38 (34)	0.521
Grade 2	18 (17.2)	21 (18.7)	0.235
Grade 3	0	2 (1.8)	0.573
**GU toxicity**			
Grade 0	27 (25.8)	25 (22.3)	0.285
Grade 1	56 (53.3)	61 (54.5)	0.594
Grade 2	18 (17.1)	21 (18.7)	0.631
Grade 3	4 (3.8)	5 (4.5)	0.432

**Table 4:** Maximum acute RTOG toxicity at 3 months of follow up

**Characteristic**	**C-Arm (%)**	**Hypo-Arm (%)**	**P value**
	**105 patients**	**112 patients**	
**GI toxicity**			
Grade 0	86 (82)	94 (84)	0.945
Grade 1	13 (12.3)	16 (14.2)	0.221
Grade 2	4 (3.8)	1 (0.9)	0.578
Grade 3	2 (1.9)	1 (0.9)	0.683
**GU toxicity**			
Grade 0	81 (77.1)	84 (75)	0.321
Grade 1	17 (16.1)	21 (19)	0.465
Grade 2	4 (3.8)	4 (3.5)	0.987
Grade 3	3 (3)	3 (2.5)	0.980

No significant correlation was found among the dosimetric and clinical parameters such as; PTV volume, rectal volume, Bladder volume, age and androgen deprivation with acute GI and GU toxicity after 3 months of follow up, as described in Table 5. In the multivariate logistic regression no variable was associated with acute GI or GU acute toxicity, as described in Table 6.

**Table 5:** Maximum acute toxicity during follow up and clinical parameters

**Characteristic**	**Grade > =2 Acute GI toxicity**		**P value**
	**C-Arm (%)**	**H-Arm (%)**	
ADT	8 (7.6)	13 (11.6)	0.103
No ADT	10 (9.5)	10 (8.9)	
PTV volume > =165	11 (10.4)	11 (9.8)	0.234
PTV volume < 165	7 (6.6)	9 (8)	
Rectal volume > = 60	9 (8.5)	11 (9.8)	0.545
Rectal volume < 60	9 (8.5)	12 (10.7)	
Age > = 70	11 (10.4)	12 (10.7)	0.756
Age < 70	8 (7.6)	8 (7.1)	
	**Grade > = 2 acute GU toxicity**		
ADT	10 (9.5)	12 (10.7)	0.645
No ADT	12 (11.4)	14 (12.5)	
PTV volume > = 165	13 (12.3)	15 (13.3)	0.453
PTV volume < 165	9 (8.5)	12 (10.7)	
Bladder volume > = 260	9 (8.5)	13 (11.6)	0.987
Bladder volume < 260	13 (12.3)	14 (12.5)	
Age > =70	11 (10.4)	14 (12.5)	0.876
Age < 70	11 (10.4)	13 (11.6)	

**Table 6:** The multivariate analysis (logistic regression) evaluating the gastrointestinal or the genitourinary toxicity > = grade 2

**Gastrointestinal toxicity**			
**Factor**	**Relative Risk**	**95% CI**	***P***
Hypofractionation treatment	1.03	0.45 - 15.56	0.953
No ADT	0.87	0.27 - 15.72	0.956
Age > = 70 years	1.29	0.68 - 13.42	0.765
PTV volume > = 165	1.33	0.72 - 11.14	0.674
Rectal volume > = 60	0.98	0.38 - 7.55	0.998
**Genitourinary toxicity**			
Hypofractionation treatment	1.17	0. 58–6.4	0.978
No ADT	1.09	0.52 – 15.65	0.743
Age > = 70 years	1.13	0.59 – 14.01	0.661
PTV volume > = 165	1.46	0.76 – 10.92	0.932
Bladder volume > = 260	0.77	0.18 – 6.92	0.839

## Discussion

The present trial is the first nonrandomized study to compare a high-dose hypofractionated with conventionally fractionated schedule using conformal radiotherapy for prostate cancer. This study was designed to test the hypothesis that a high- dose hypofractionation regimen is equivalent to a conventional fractionation scheme in terms of acute GI and GU toxicity. This hypothesis was determined from the assumption that the α/β ratio would be 1.5–2 Gy for prostate cancer [3,4] and 10 and 3 Gy for early and slowly proliferating normal tissue, respectively. With the delivery of the same equivalent total dose to prostate tumors using a hypofractionation regimen, the corresponding equivalent doses to normal tissue would be lower. Therefore, with a slight prolongation of the shorter overall treatment time (from 4.5 weeks), both acute and late toxicity would be reduced compared with that occurring after conventional fractionation.

Based on this premise, this radiation protocol has been used at our institute since 2009. It was also empirically designed to provide acceptable biochemical control with satisfactory levels of toxicity, being convenient for the patients and with advantages to the hospital in terms of time and resource management. To date, the dose used in this trial is one of the highest BED delivered in a hypofractionated external beam regimen, as demonstrated in Table 7[9,10,12-15]. Our results demonstrate that 69 Gy delivered in 23 daily fractions over 4.5 weeks is well tolerated using our RT technique and dose constraints, as seen in Table 7.

**Table 7:** Overview of published clinical data on hypofractionation in prostate cancer

**References**	**Fractions**	**Fraction size**	**Total dose**	**weeks**	**NTD2 for α/β**			**Acute toxicity**	
					**α/β = 1.5**	**α/β =3**	**α/β =10**	**GI (%)**	**GU (%)**
Livsey et al. [12]	6	6	36	3	77.1	64.8	48		
Kupelian et al. [9]	28	2.5	70	5.5	80	77	72.9	19	15
Soete et al. [15]	16	3.5	56	4	80	72.8	63	38	39
Madsen et al. [10]	5	6.7	33.5	1	78.5	65	46.6	22	13
Pollack et al. [13]	26	2.7	70.2	5	84.2	80.5	74.3	18	48
Martin et al. [14]	20	3	60	4	77.1	72	65	12	25
FAMEMA trial (present study)	23	3	69	4.5	88.7	83	76	20.5%	23.2%

Abbreviations: GI = gastrointestinal;GU = genitourinary; NTD2 = Equivalent dose (Gy) for 2-Gy fractionation; BED = biological effective dose.

Moreover, the hypofractination schedule was quite well tolerated with more than 45% of patients presenting no acute GI toxicity during the treatment and at 3 months of follow up, only 2% of patients had residual grade > = 2 toxicity. This data are comparable to other hypofractionation cohorts with residual Grade > =2 GI toxicity rates of 4–5%. The incidence of Grade 2 or greater acute reactions reported in the present report for our standard fractionation was not greater (rectal 17.2%, urinary 20.9%), than other trials of dose escalation. Acute toxicity has been addressed in several randomized clinical trials of dose escalation using standard fractionation. In a French trial [16], 30% of patients presented with acute rectal reactions of Grade 2 or greater and 37% with urinary reactions of Grade 2 or greater in the arm treated to a mean dose of 78.5 Gy. In another study from The Netherlands [17], gastrointestinal complications of Grade 2 or greater were experienced by 51% of patients in the 78-Gy arm and urinary complications of Grade 2 or greater in 55%. Therefore, the use of five fractions weekly, instead of three or four, with an overall treatment time of 38 days for our hypofractionation regimen, have not increased the acute toxicity.

Another point that deserves attention is our margin given to the planning target volume (PTV). Despite the use of a larger posterior margin (7mm) given to the PTV in our cohort, the rates of acute GI toxicity were similar to Soete et al. [15] delivering 56 Gy in 16 fractions of 3.5 Gy with 3 mm of margin. In their study was reported 5% of Grade 2 acute GI toxicity with no Grade 3 toxicity. For us, this satisfactory level of acute toxicity observed in our study can be result of an accurate treatment planning and set up verification, associated with a close attention to the dose–volume constraints for the organs at risk (Table 1). Although, does not exist a consensus on the optimal dosimetric parameters to be used in clinical practice. Rectal and bladder constraints are used to reduce the incidence of bladder and rectal toxicity. So, we speculate that although the dose–volume histograms based on the initial planning CT may not reflect the real dose received by the rectum because of displacement of the prostate and rectum during and between treatments, the use of restrictive DVH can help to maintain the acute toxicity rates in a satisfactory level.

In our univariated and multivariated analysis comparing dosimetric and clinical variables (e.g., risk group designation, PTV volume, SV irradiation, or hormonal therapy) between the two groups, no significant relationship with acute GI or GU effects was observed. This can be probably related to the large variability of bladder and rectum volumes during and between the treatments. This founds are in agreement with those of Pollack et al. [13], who also did not observe a target volume or normal tissue dose–volume dosimetric relationship with either acute GI or GU toxicity, nor with group designation or use of hormonal therapy.

## Conclusion

We present here our early outcomes for a high dose hypofractionated conformal radiotherapy regime for the treatment of prostate cancer. This study suggests that hypofractionated dose-escalated radiotherapy using conformal radiotherapy in prostate cancer is feasible and produces acceptable toxicity with the dose constraints used. No difference in incidence or severity of acute gastrointestinal and genitourinary toxicity was observed when compared to conventional fractionation. Moreover, the acute toxicity rates were comparable to those of other dose escalation trials using standard or hypofractionated schedules, being transient, with only 2 patients having persistent Grade 2 or higher GI toxicity by 3 months follow up.

## Competing interests

The author & co-authors have no conflicts of interest to declare.

## Authors’ contributions

VGA, DsLBG and SEJ performed the statistical analysis and wrote the paper. DsBB, CYB, MVS, FRJR, PMC, RBT, ZGC and ES collected the data. All authors read and approved the final manuscript.
